# zDALY: An adjusted indicator to estimate the burden of zoonotic diseases

**DOI:** 10.1016/j.onehlt.2017.11.003

**Published:** 2017-11-28

**Authors:** Paul R. Torgerson, Simon Rüegg, Brecht Devleesschauwer, Bernadette Abela-Ridder, Arie H. Havelaar, Alexandra P.M. Shaw, Jonathan Rushton, Niko Speybroeck

**Affiliations:** a Section of Epidemiology, Vetsuisse Faculty, University of Zurich, Winterthurerstrasse 270, CH-8057 Zurich, Switzerland; b Institute of Health and Society (IRSS), Université catholique de Louvain, Clos Chapelle aux champs, 30, 1200 Bruxelles, Belgium; c Department of Public Health and Surveillance, Scientific Institute of Public Health (WIV-ISP), Brussels, Belgium; d Department of Food Safety and Zoonoses (FOS), World Health Organization, Avenue Appia 20, CH-1211 Geneva 27, Switzerland; e Institute for Sustainable Food Systems, Emerging Pathogens Institute and Animal Sciences Department, University of Florida, Gainesville, FL, USA; f Institute for Risk Assessment Sciences, Faculty of Veterinary Medicine, Utrecht University, Utrecht, The Netherlands; g Division of Infection and Pathway Medicine, Edinburgh Medical School: Biomedical Sciences, College of Medicine and Veterinary Medicine, The University of Edinburgh, Chancellor's Building, 49 Little France Crescent, Edinburgh EH16 4SB, UK; h A P Consultants, 22 Walworth Enterprise Centre, Duke Close, Andover SP10 5AP, UK; i Institute of Infection and Global Health, University of Liverpool, UK

**Keywords:** Disability-adjusted life years, Zoonotic diseases, Societal impact, Burden of disease, Animal disease losses

## Abstract

The burden of human diseases in populations, or for an individual, is frequently estimated in terms of one of a number of Health Adjusted Life Years (HALYs). The Disability Adjusted Life Year (DALY) is a widely accepted HALY metric and is used by the World Health Organization and the Global Burden of Disease studies. Many human diseases are of animal origin and often cause ill health and production losses in domestic animals. The economic losses due to disease in animals are usually estimated in monetary terms. The monetary impact on animal health is not compatible with HALY approaches used to measure the impact on human health. To estimate the societal burden of zoonotic diseases that have substantial human and animal disease burden we propose methodology which can be accommodated within the DALY framework. Monetary losses due to the animal disease component of a zoonotic disease can be converted to an equivalent metric using a local gross national income per capita deflator. This essentially gives animal production losses a time trade-off for human life years. This is the time required to earn the income needed to replace that financial loss. This can then be assigned a DALY equivalent, termed animal loss equivalents (ALE), and added to the DALY associated with human ill health to give a modified DALY. This is referred to as the “zDALY”. ALEs could also be estimated using willingness-to-pay for animal health or survey tools to estimate the replacement time value for animals with high societal or emotional value (for example pets) that cannot be calculated directly using monetary worth. Thus the zDALY estimates the impact of a zoonotic disease to animal and human health. The losses due to the animal disease component of the modified DALY are straightforward to calculate. A number of worked examples such as echinococcosis, brucellosis, Q fever and cysticercosis from a diverse spectrum of countries with different levels of economic development illustrate the use of the zDALY indicator.

## 1 Introduction

An understanding of the economic and social impacts of diseases is central to the decision making process for disease control. Animal disease impact is widely studied using economic models based on monetary costs [1], [2], [3]. Therefore animal losses can be estimated for the values of livestock and livestock products lost through diseases [4], [5]. In health economics the cost of human disease can be quantified in terms of cost of treatment and loss of income during convalescence in cost of illness studies [6], [7]. For example in the Netherlands the cost of illness for 14 food-related pathogens for 2011 was estimated at approximately €468 million [8]. These costs included several zoonoses such as toxoplasmosis, which had costs of approximately €55 million.

The calculation of a monetary burden of human ill health, to incorporate death and disability as well as the cost of illness, has been criticised for a number of reasons. There is no standard methodology to calculate the monetary value of life (or loss due to premature death) resulting in great variability amongst different studies [9]. This also varies with per capita income, with the lives of individuals from high income countries being assigned, on average, a greater monetary value. Indeed, this has been modelled to estimate the statistical value of life with gross domestic product per capita as a covariate [10]. Likewise, costs of medical treatment and loss of wages are usually higher in upper than in lower income countries. This could perversely suggest that the greatest economic impact of disease is in wealthy countries, despite having higher life expectancies and lower disease incidence than low income countries. Thus estimations of the monetary cost of disease effectively assign higher values to equivalent health outcomes in higher income countries than to the same outcomes in the low income countries [11].

Because of these and the wider issues around valuing human life, non-monetary population health metrics such as disability adjusted life years (DALYs) have been developed to quantify the burden of premature death and disease and injury [12]. DALYs are calculated by adding the number of years lived with disability adjusted for the severity of the disease (YLDs) and the number of years of life lost due to premature mortality (YLLs) [13]. DALYs have been adopted by the WHO to quantify the global burden of disease [14] and the global burden of foodborne diseases [15]. They can also be used in cost-effectiveness studies such as cost per DALY averted, for example rabies in Kazakhstan [16].

In the animal health field, there have been attempts to mirror these burden estimates for animal diseases. There are important conceptual difficulties: how to compare within and across animal species, the fact that for livestock producers maximising their animals' life expectancy is not necessarily a goal and that the same physical disabilities have very different outcomes in different livestock productions systems. Work sponsored by the World Bank [17] assigned livestock unit (LSU) values to different animal species, similar to the standard approach based on animal weights and mapped the total units to be lost through death, destruction or slaughter. Despite emphasizing the unevenness of and major gaps in the data available on livestock disease impact, this study did illustrate the relative geographical burden of fatal animal diseases. However, it assigned the same weight to animals of a single species across the world and did not quantify non-fatal outcomes – the production losses which undermine the profitability of livestock keeping in many situations. For example for echinococcosis, there are no reports of losses in China, India and much of the Middle East and North Africa in the World Bank Atlas yet these regions are highly endemic for *Echinococcus granulosus* [18]. By contrast, the well-established methods of animal health economics [3], allow for the impacts of animal disease, including trade effects and the cost of mitigating measures to reduce physical losses, to be estimated in livestock across production systems and regions, and aggregated into a single currency. By using willingness-to-pay and other household survey methods, the monetary value of companion animals and wildlife can be estimated in a range of contexts. As well as owners' valuations, financial awards in legal cases are often linked to the lifetime cost of keeping companion animals and for wildlife, other monetary considerations include their commercial contribution to tourism.

As a non-monetary value, DALYs do not capture other societal burdens that may not be directly human health-related. With zoonotic diseases there is clearly potential for a human disease and an animal disease burden, as by definition zoonoses infect both animals and humans [19]. Presently there has been no satisfactory metric developed that can incorporate both these burdens and estimate their relative share in the societal cost of disease. For zoonoses control projects, cost sharing in proportion to expected disease control benefits has been proposed [20], but this is related only to the estimated monetary benefits in terms of animal disease prevention and monetary savings from reduced cost of human illness, which are assessed separately from the DALY component. To be incorporated into the DALY metric, livestock deaths and production losses, need to be quantified into an animal disease burden metric which can stand alongside the DALY. Such a metric should reflect the impact of the animal disease on its owner in terms of the time that might be required to replace that animal or recoup the losses caused by its illness. Thus a subsistence farmer in sub-Saharan Africa who loses a cow, of value $500, due to disease may suffer a much greater impact than a farmer from an industrialized nation who might lose a highly productive cow of value $1000. If the former has an income of just $1000 per annum and the latter $40,000 per annum, the relative time in terms of income generation to replace that loss would be six months for the former and 1.3 weeks for the latter. The same approach can be extended to production losses from non-fatal animal diseases: if the same cow on a sub-Saharan African farm were to be infected with brucellosis it might suffer a 15% reduction in milk production and fertility [21] equivalent to, say, $75. In this case the African farmer would need nearly 4 weeks to replace that loss, whereas a farmer in an upper income country would require less than 1.5 days to replace a similar proportional loss.

These impacts can thus be viewed as time that the livestock owner has been forced to sacrifice to replace the loss of his animal or to make up for the reduced output that a non-fatal animal disease causes. In this manuscript we propose a modified DALY for zoonotic disease, termed zDALY, that has an additional component, termed animal loss equivalents (ALE). The ALE can be estimated from the monetary value of livestock losses and local per capita income by using a time trade-off approach to estimate an equivalent burden to the human population. The clear rationale is therefore to incorporate animal health losses into the DALY framework to assess the burden of diseases that transmit between animals and humans. In the DALY framework, non-fatal human illnesses and injuries are given a disability weight (DW) and the duration of this DW gives an equivalent number of lives lost and is essentially a time trade-off by assigning the time lived with the disability to a shorter time of healthy years (i.e. lived without the disability). It is proposed that the ALE component in the zDALY is also given a time equivalent of healthy years. A number of worked examples on different zoonotic diseases from low, middle and upper income countries are used to illustrate this concept.

## 2 Materials and methods

### 2.1 Data

#### 2.1.1 Cystic echinococcosis

Human cystic echinococcosis (CE) is caused by the larval stage of *E. granulosus*. This results in a space occupying lesion, usually in the liver or lungs but occasionally affecting other organs such as the central nervous system. Most species of farm livestock can be infected with high prevalences frequently seen in sheep. CE can result in a substantial human disease burden and have a substantial economic impact on animal productivity [22], [23]. In animals these include condemnation of edible offal, a lowered food conversion ratio and a decreased milk yield. The monetary value of these effects is used to estimate the ALE component of the zDALY. Both humans and livestock become infected by contact with infected dogs which are the definitive hosts of *E. granulosus* [24]. There are a number of estimates of the impact of CE in various countries including Iran [25], Jordan [26], Kyrgyzstan [27], Peru [28], Spain [29] and Tunisia [30]. The raw data, converted to 2015 US$ are presented in Table 1.

**Table 1:** Data used to estimate the zDALY for cystic echinococcosis (CE).

	Year	Animal losses^a^	US$ 2015 equivalent	GNI per capita 2015	Treatment-seeking human cases/annum	Non treatment-seeking human cases/annum
Iran	2010	$132 million	$143 million	$6550	922	937
Jordan	2001	$3.58 million	$4.82 million	$4680	187	200
Kyrgyzstan	2013	$5.5 million	$5.6 million	$1170	884	1226
Peru	2007	$3.85 million	$4.40 million	$6130	2890	4380
Spain	2005	€15.5million	$25.8 million	$28,530	208	208
Tunisia	2000	$8.38 million	$11.45 million	$3980	1339	1127

a As reported in [25], [26], [27], [28], [29], [30].

#### 2.1.2 Brucellosis

Human brucellosis is a zoonotic bacterial disease caused by a number of species of the genus *Brucella*. Human infection can result in intermittent fever, headaches, weakness, arthralgia, myalgia, weight loss, orchitis and spontaneous abortion [31]. In animals, brucellosis can cause a variety of clinical manifestations, particularly abortion, long run effects on fertility and reduced milk yield, resulting in substantial economic losses. These make up the ALEs contributing to the zDALY. Transmission to humans occurs through the consumption of infected, unpasteurized milk products, through direct contact with infected animal parts and through the inhalation of infected aerosolized particles [32]. Data on human brucellosis are officially reported figures from the Kyrgyz Ministry of Health [33]. Data for the economic impact of brucellosis in animals are based on Zinsstag et al. [21].

#### 2.1.3 Q fever

Q fever is caused by the bacterium *Coxiella burnetti*. Sheep, goats, and cattle are the primary reservoirs. The most common signs of Q fever in animals are abortion during late pregnancy or weak offspring [34]. This results in the economic losses in affected animals used to estimate ALEs and hence contribute to the zDALY. Infection of humans usually occurs by inhalation of bacteria or from ingestion of unpasteurized dairy products. Humans present with mild flu-like symptoms with occasional complications such as endocarditis. There was an epidemic of Q fever in the Netherlands with cases peaking in 2009 [35]. The estimated economic costs of the outbreak and the burden of disease in terms of DALYs have been estimated [34], [36].

#### 2.1.4 Cysticercosis

*Taenia solium* causes cysticercosis in pigs and neurocysticercosis in humans. The former can lead to substantial livestock losses whilst the latter can result in a number of neurological syndromes such as epilepsy [37]. Human cysticercosis results from faecal-oral contamination with *T. solium* eggs from tapeworm carriers [38]. The dual monetary impact to animal production and human health burden in terms of DALYs has been estimated for West Cameroon [39]. Here it was assumed that infection with cysticercosis reduced the value of pigs by 30% and this was the basis for estimating the ALE component of the zDALY. The data from this report are used to estimate the number of zDALY due to *T. solium* cysticercosis.

### 2.2 zDALY

To calculate the zDALY, information on the incidence of human disease of interest together with the natural history of the disease and the morbidity and mortality rates are required. The degree of morbidity is required to estimate the DWs and the duration to estimate the YLDs [40]. Numbers of fatal cases, stratified by age are required for the YLLs. For the ALE component information about the morbidity and mortality in livestock or other domestic animals is required in addition to the local value of these animals and their products. The income per head of the human population is also necessary to estimate a time trade-off for an economic loss due to animal disease. For animals that have no obvious commercial value, household survey methods, such as willingness-to-pay, and other approaches discussed above, could be used to estimate the time trade-off equivalent.

The human burden of disease was calculated as DALYs. Incidence-based YLDs were calculated by multiplying the numbers of cases of disease reported by their duration and DW. The YLLs were calculated as the numbers of deaths and their residual life expectancy at the time of death. Suitable DWs from the GBD 2010 study [41] were used in all estimates. To calculate the ALEs, first an estimate of the annual monetary impact of the animal diseases, including production losses, was made. These estimates were already published previously in several of the datasets used, but were updated to 2015. This was achieved by converting to US$ at the prevailing exchange rate at the time of the study (http://www.x-rates.com/historical) followed by a correction for the US$ inflation rate since the date of the estimates (http://www.usinflationcalculator.com/). This total monetary impact of the animal disease was then divided by the gross national per capita income (GNI) to obtain ALEs at 2015 values.

ALE = monetary value of animal health losses/GNI per person.

In all our calculations we used nominal values converted to US$ at the mean exchange rate in 2015 to make the estimates of ALEs. Nominal values were selected, as all the studies used current rather than international dollar or local currency values.

Thus zDALY = YLL + YLD + ALE.

GNI per capita data at current dollars were according to the World Bank figures (downloaded from http://data.worldbank.org/indicator/NY.GNP.PCAP.CD).

## 3 Results

**Fig. 1:**
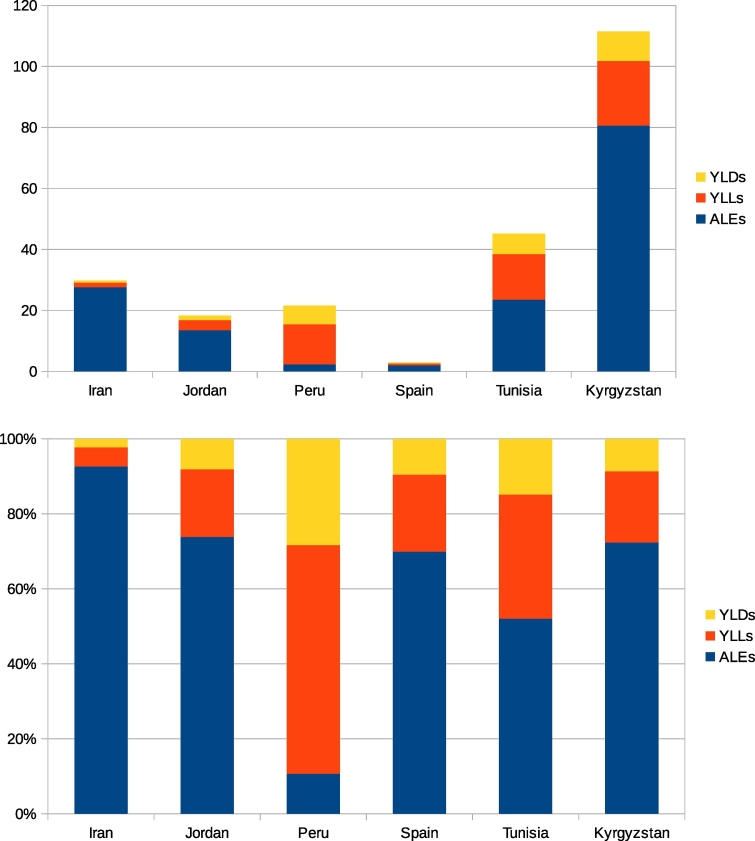
Top: Estimated number ofzDALYs per 100,000 population for echinococcosis in various countries. Bottom: proportion of zDALY contributed by YLDs, YLLs and ALEs.

Table 2 gives the estimated ALEs for echinococcosis in different countries, whilst Table 3 gives the DALYs and incident zDALYs. The relative contribution to the zDALY from DALYs and ALEs varies between different countries. For echinococcosis in Iran for example, most of the burden is from the livestock sector. In Peru the opposite is the case. This is illustrated in Fig. 1. The monetary losses due to echinococcosis vary from $4.4 million/year for Peru, to $143 million/year for Iran (Table 1). However, when adjusted for purchasing parity and converted into ALEs, Spain, for example, incurs just 904 ALEs, compared to Kyrgyzstan which incurs 4795 ALEs (Table 2). This is despite the fact that the gross economic loss in Spain is $25.8 million, some four times that of Kyrgyzstan of $5.5 million (Table 1).

**Table 2:** Animal loss equivalents (ALEs): CE.

	Human population	ALEs	ALEs/100,000 person years
Iran	79.1 million	21,832	27.6
Jordan	7.59 million	1030	13.5
Kyrgyzstan	5.94 million	4786	80.6
Peru	31.8 million	718	2.3
Spain	46.1 million	904	2.0
Tunisia	11.3 million	2877	25.5

**Table 3:** Burden of disease for CE in terms of DALYs and zDALYs.

	YLDs	YLLs	DALYs	DALYs/100,000 person years	ALEs/100000 person years	zDALY/100,000 person years
Iran	537	1198	1735	2.19	27.6	29.8
Jordan	113	250	363	4.78	13.5	18.3
Kyrgyzstan	573	1258	1831	30.8	80.6	111
Peru	1943	4185	6128	19.3	2.3	21.6
Spain	126	271	397	0.86	2.0	2.86
Tunisia	758	1689	2447	21.7	25.5	47.2

**Fig. 2:**
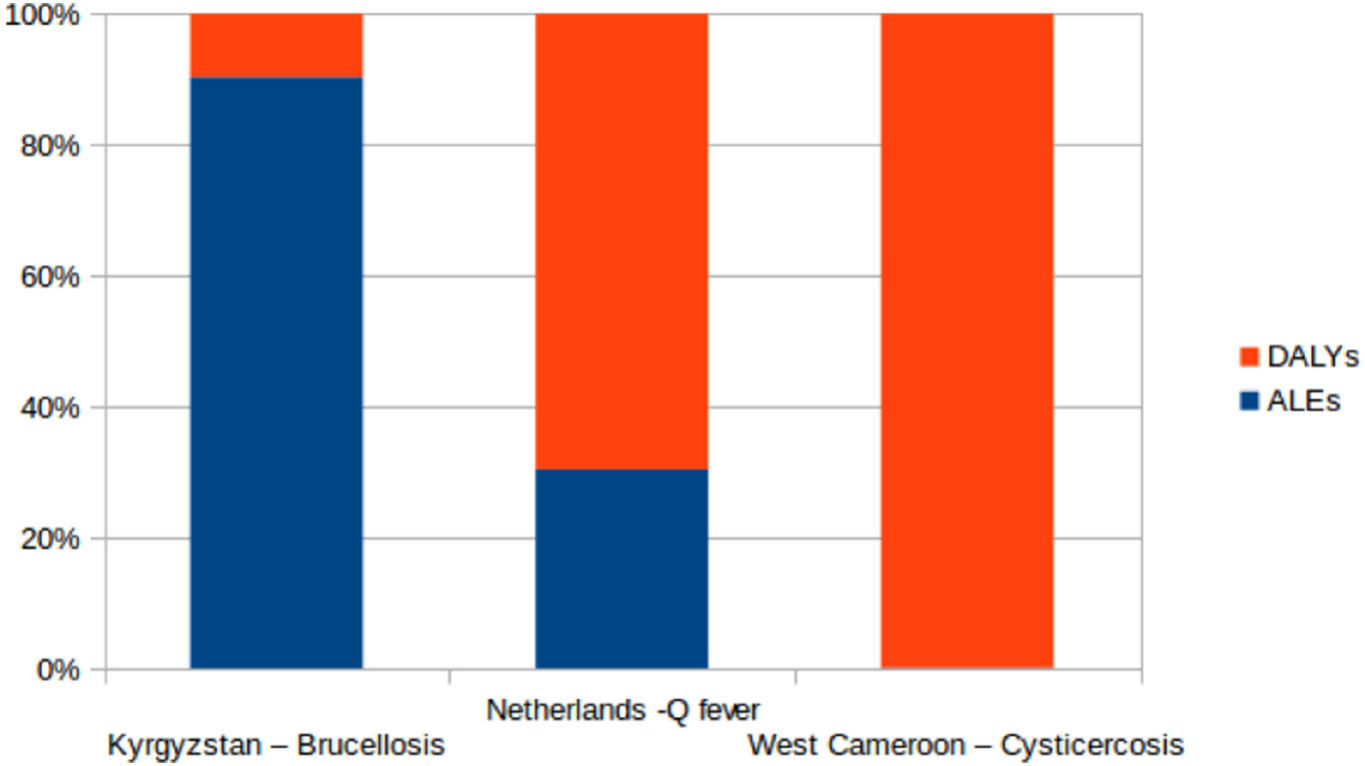
Proportion of zDALY contributed by the standard DALY and ALE by brucellosis in the Kyrgyz Republic, Q fever in the Netherlands and cysticercosis in West Cameroon.

For brucellosis in Kyrgyzstan 90% of the societal burden is from animal losses (Table 4, Fig. 2). This compares to about 31% for Q fever in the Netherlands, but under 1% for cysticercosis in West Cameroon. In the Netherlands, Q fever had a monetary impact on animal health of approximately $122 million. This was more than ten times higher than the monetary impact of brucellosis in livestock in Kyrgyzstan. However, in terms of ALEs, there was a much higher impact in Kyrgyzstan with 10,085 ALEs (170 per 100,000 people) compared to 2537 (15 per 100,000 in the Netherlands).

**Table 4:** Burden of other diseases.

Country	Disease	Animal losses	US$ 2015 equivalents	Human cases	DALYs	DALYs/100,000 person years	ALEs	ALEs/100,000 person years	zDALYs/100,000 person years
Kyrgyzstan +	Brucellosis	$10 million	$11.8 million	3350	1075	18	10,085	170	188
Netherlands^a^	Q fever	€85million	$124 million	4107	5797	34	2537	15	49
West Cameroon^b^	Cysticercosis	€478.838	$794.842	18,268	45,838	9050	602	12	9061

+ DALY estimate from FERG data [42] (DALYs per case).

a Q fever outbreak – data from [34], [35], [36], duration of outbreak 2007–2011.

b Data from [39].

## 4 Discussion

DALYs reflect only the importance of human disease or injury to society. Zoonotic diseases can have adverse outcomes for both human and animal subjects. We have proposed the zDALY to quantify this. This also takes into account time lost due to animal morbidity and mortality. As for the DALY, our focus in this quantification of both human and animal losses is on the impact of morbidity and mortality: we do not consider the wider prevention and treatment costs of ill health in people and animals and we restrict the burden of human disease to the DALY component. The essential component to include animal health losses is converting monetary losses due to animal disease into time lost to society. This is achieved by equating it to the time needed to earn the income to recover that loss. Time trade-offs are one method used to estimate the value of health states when calculating Quality Adjusted Life Years (QALYs) [43]. Therefore the same concept could indeed be applied to QALY losses. However our focus is on the DALY as this is the most common HALY metric for quantifying burden of disease. Also, DALYs were originally formulated from a disease perspective, while QALYs were formulated from a patient perspective. As the addition of livestock losses is proposed for quantifying the disease burden of specific diseases, the perspective coincides with that of the DALY.

Although time trade-offs have been used to estimate DWs for DALYs [44], [45], in GBD 2010 DWs for DALY calculations were developed through household surveys. These asked respondents paired comparison questions, in which respondents considered two hypothetical individuals with different, randomly selected health states and indicated which person they regarded as healthier. A further survey included questions about population health equivalence for a subset of 30 health states. In these questions, respondents were asked to compare the health benefits of different life-saving or disease-prevention programmes to anchor disability weights to the necessary scale (from zero to one) [41]. It would also be possible to undertake similar household surveys to determine equivalences of monetary losses with health states, but these would need to be controlled for local income levels. Furthermore, we use monetary values of livestock losses purely as a technique to estimate the time trade-off to calculate the ALE. Some animals such as a treasured domestic pet or an animal of great religious significance may have a value to the owner far greater than its commercial worth. Such issues can still be addressed by household surveys to gauge how these are valued. The ALE could then be calculated for the respective species based on the results of such surveys.

The strength of our approach is that it values different monetary losses in terms of their local impact. For example, in Spain the monetary loss due to echinococcosis is approximately $56,000 per 100,000 person years. In Peru it is $14,000 per 100,000 person years. However, because Peru has a considerably lower per capita income, there is a higher burden of ALEs in Peru (2.3 per 100,000 person years) compared to Spain (2.0 per 100,000 person years). Thus ALEs have the desirable property of being linked to the actual impact of the losses felt to the population. It may be that ultimately an ALE is considered to be of less value than a whole life year – a person may value their working time at say, only half their time. Such equivalences could be further explored and nuanced once the concept of the ALE has gained some traction. A potential limitation of our approach is that for the ALE component our examples examine direct losses due to mortality and morbidity (converted to a time trade-off) and there are other aspects, such as intervention costs. However, it thus remains analogous to estimating DALYs where costs of treatment or control are not included in the metric. Therefore comparable and analogous data must be used for estimating the ALE and DALY components of the zDALY.

There have been attempts to put a monetary value on a DALY [46]. In the context of zoonotic disease, we propose the opposite by giving a local monetary loss due to livestock disease an equivalent DALY. It may be possible to extend this approach to estimating the impact of animal diseases that are not zoonoses. An example would be the foot and mouth disease (FMD) outbreak in the United Kingdom in 2001. Over 6 million animals were slaughtered resulting in approximately £3.1 billion ($4.5 billion) losses to agriculture and the food chain [47]. The GNI per person in the UK in 2001 was $26,300, so the impact in terms of ALEs would be approximately 170,000. This considerable number of DALY equivalents would reflect the devastation caused to UK agriculture during the outbreak. Another possible extension of the approach would be to set it in a wider One Health framework, looking at ecosystem impacts, such as the time equivalent required for an ecosystem to recover from damage.

The zDALY can, like the DALY, be used in priority-setting across sectors. In cost-effectiveness studies the cost per zDALY averted can be used to compare different interventions for the same disease, or more broadly to compare the cost-effectiveness of control compared to the cost-effectiveness of other competing public health projects. It may improve the efficiency of resource allocation as the zDALY accounts for the often substantial societal impact to animal health of zoonotic diseases. This is particularly true in marginalised communities which subsist on small numbers of livestock. In poor countries, diseases kill many animals. In 2009 a total of 2.1 million cattle and 13.1 million sheep died of disease in just three sub-Saharan African countries: Burkina Faso, Mali and Niger. This represented a direct loss of 5.5% of total GDP [48]. Losses from other livestock and indirect losses would further substantially inflate this figure. Furthermore, half of the world's hungry are smallholder farmers who depend on agriculture for their livelihood [49]. Thus the loss due to disease of an agricultural animal can have a devastating effect on such smallholders. The DALY does not take this into account, as the metric only analyses the burden of human disease. Thus the zDALY could be a valuable tool in public health economics priority setting. For example, data from the GBD 2010 study would ideally “be supplemented with additional information regarding the impact of different conditions on the health and welfare of individuals in different locales” [50]. The zDALY provides such information on the impact of animal disease and could also potentially drive sustainable development action. By anchoring disease losses firmly in relation to DALYs for people and in terms of local monetary losses for animals this joint metric is clear and transparent to both the animal and human health constituency, while providing an aggregate metric of the relative losses incurred.

In 2011, the Disease Reference Group on Zoonoses and Marginalised Infectious Diseases (DRG6), convened by the Special Programme for Research and Training in Tropical Diseases (TDR), identified nine macro research priorities related to zoonotic diseases of marginalised populations [51]. One of these priorities was the development of a comprehensive methodology for calculating the societal burden of disease attributable to zoonoses. The zDALY metric provides a straightforward, practical and rigorous answer to this need.
